# Case Report: Etoposide-nedaplatin induced rhabdomyolysis in a small cell lung cancer patient

**DOI:** 10.3389/fphar.2023.1214149

**Published:** 2023-08-22

**Authors:** Xiaohu Xu, Xiao Wu, Mingmin Zhang, Qi Wang

**Affiliations:** Department of Integrated Traditional Chinese and Western Medicine, Tongji Hospital, Tongji Medical College, Huazhong University of Science and Technology, Wuhan, China

**Keywords:** rhabdomyolysis, etoposide, nedaplatin, adverse reactions, case report

## Abstract

Rhabdomyolysis syndrome refers to the breakdown and necrosis of muscle tissue due to various reasons and caused by the release of intracellular contents into the blood stream, which can lead to acute renal failure or even death. In this article, we describe for the first time a case report of severe rhabdomyolysis induced by etoposide-nedaplatin chemotherapy in a small cell lung cancer (SCLC IIIb) patient. The patient developed progressive general muscle pain and weakness after the first cycle of chemotherapy, accompanied by elevated creatine kinase (CK), myoglobin (Mb), alanine aminotransferase (ALT), spartate aminotransferase (AST), and lactate dehydrogenase (LDH). Examination of and inquiry regarding the medical history were used to exclude various factors of rhabdomyolysis caused by trauma, strenuous activities, infections, drugs, hyperthermia, and immunity; the patient was diagnosed with severe rhabdomyolysis induced by chemotherapy. After treatment with intravenous fluids and methylprednisolone, the patient’s symptoms were relieved and laboratory results were significantly improved. An unexpected situation arose, in that the lung CT scan showed that the lung mass was significantly smaller than that before chemotherapy; the reason for this is not clear. Rhabdomyolysis induced by anti-cancer drugs, especially chemotherapy drugs, is rarely reported and easily overlooked. Therefore, physicians should be aware of this rare but potentially serious complication when using chemotherapy drugs.

## Introduction

Rhabdomyolysis syndrome refers to the breakdown and necrosis of muscle tissue due various reasons and caused by the release of intracellular contents into the blood stream. It is characterized by muscle weakness, pain, and dark tea–colored urine (Cabral et al., 2020). Laboratory tests show that serum creatine kinase (CK) is elevated, which is an important basis for the diagnosis of rhabdomyolysis. Rhabdomyolysis is generally considered to be diagnosed when CK is at least five times the normal level or >1,000 U/L. Severe rhabdomyolysis can lead to acute renal failure or even death. It usually results from trauma, strenuous activities, infections, hyperthermia, inherited enzyme deficiencies, and myopathies. Medications such as lipid-reducers, antidiabetics, psychiatrics, bisphosphonates, or antimicrobials are important causes of rhabdomyolysis (Cetrone et al., 2014; Maqoud et al., 2021; Scala et al., 2022). However, rhabdomyolysis induced by anti-cancer drugs, especially chemotherapy drugs, is rarely reported. In this article, we describe for the first time a case report of severe rhabdomyolysis induced by etoposide-nedaplatin chemotherapy in an SCLC patient.

Drug-induced rhabdomyolysis is not a common problem of chemotherapy drugs; however, it is easily overlooked by physicians. Most cases can be improved after immediate treatment; nevertheless, symptoms of fatigue and pain can still remain, which seriously affect the quality of life. This reminds physicians to be aware of this rare but potentially serious complication when using chemotherapy drugs, although the cause of adverse reactions induced by anti-tumor drugs is still unclear.

## Case report

A 71-year-old male was admitted to Tongji Hospital, Tongji Medical College, Huazhong University of Science and Technology on 9 April 2022, and complained of chest tightness, wheezing, and coughing for more than 3 months. A biopsy and brushing were performed at the upper left lobar bronchial opening by electronic bronchoscopy on 12 April 2022. The histopathological diagnosis was small cell lung carcinoma (SCLC IIIb). Immunohistochemistry showed PCK (weak+), CK8/18 (weak+), CD56 (+), Syn (partially weak+), CgA (partially weak+), INSM1 (partially+), NKX2.2 (partially weak+), SSTR2 (−), TTF-1 (−), RB1 (+), CD20 (−), CD20 (positive control+), CD3 (2V6) (−), LCA (−), P53 (diffuse strong+, suggesting mutation), and Ki-67 (LI approximately 90%).

After the patient provided written informed consent, he received the first cycle of EP (etoposide, 100 mg/m^2^, D1–D3 + nedaplatin, 80 mg/m^2^, D1) regimen chemotherapy starting on 20 April (as Day 1). On 22 April (Day 3), the patient developed chest tightness, weakness, wheezing, palpitation, increased blood pressure, and decreased oxygen saturation; therefore, chemotherapy on the third day was suspended. He underwent a lung enhanced CT examination that showed a mass shadow in the left upper lobe, a neoplastic lesion with involvement of the left pulmonary artery with atelectasis in the left upper lobe, micro-nodules in the upper lobe of the right lung and the lower lobe of both lungs, left pleural effusion, left lung insufficiency, aortic wall calcification, and gallstones. An electrocardiogram revealed sinus bradycardia and frequent ventricular premature beats. On 24 April (Day 5), color doppler echocardiography showed no abnormal heart structure or valve activity. The symptoms improved after treatment with oxygen inhalation and cardio-protective therapy; however, the patient refused to continue chemotherapy and was discharged on 27 April (Day 8).

After discharge, the patient developed a general feeling of fatigue, which gradually worsened over the next week so that he could not stand up and walk on his own. This was accompanied by shoulder pain that gradually developed into a general feeling of muscle pain and tightness of the four limbs. On 21 May (Day 32), he was admitted to a local hospital for emergency treatment. Laboratory tests showed creatine kinase (CK) 11,002 U/L (normal range 59–248 U/L), myoglobin (Mb) > 2,000 ng/mL (normal range ≤154.9 ng/mL), alanine aminotransferase (ALT) 165 U/L (normal range ≤41 U/L), aspartate aminotransferase (AST) 518 U/L (normal range ≤40 U/L), and lactate dehydrogenase (LDH) 846 U/L (normal range 135–225 U/L). After rehydration and diuretic treatment, the levels of serum CK, Mb, ALT, AST, and LDH were markedly elevated to 14,947 U/L, >2,000 ng/mL, 325 U/L, 1,146 U/L, and 1,236 U/L, respectively, on 24 May (Day 35). Physicians at the local hospital recommended that the patient be transferred to the higher-level hospital for further diagnosis and treatment.

**FIGURE 1:**
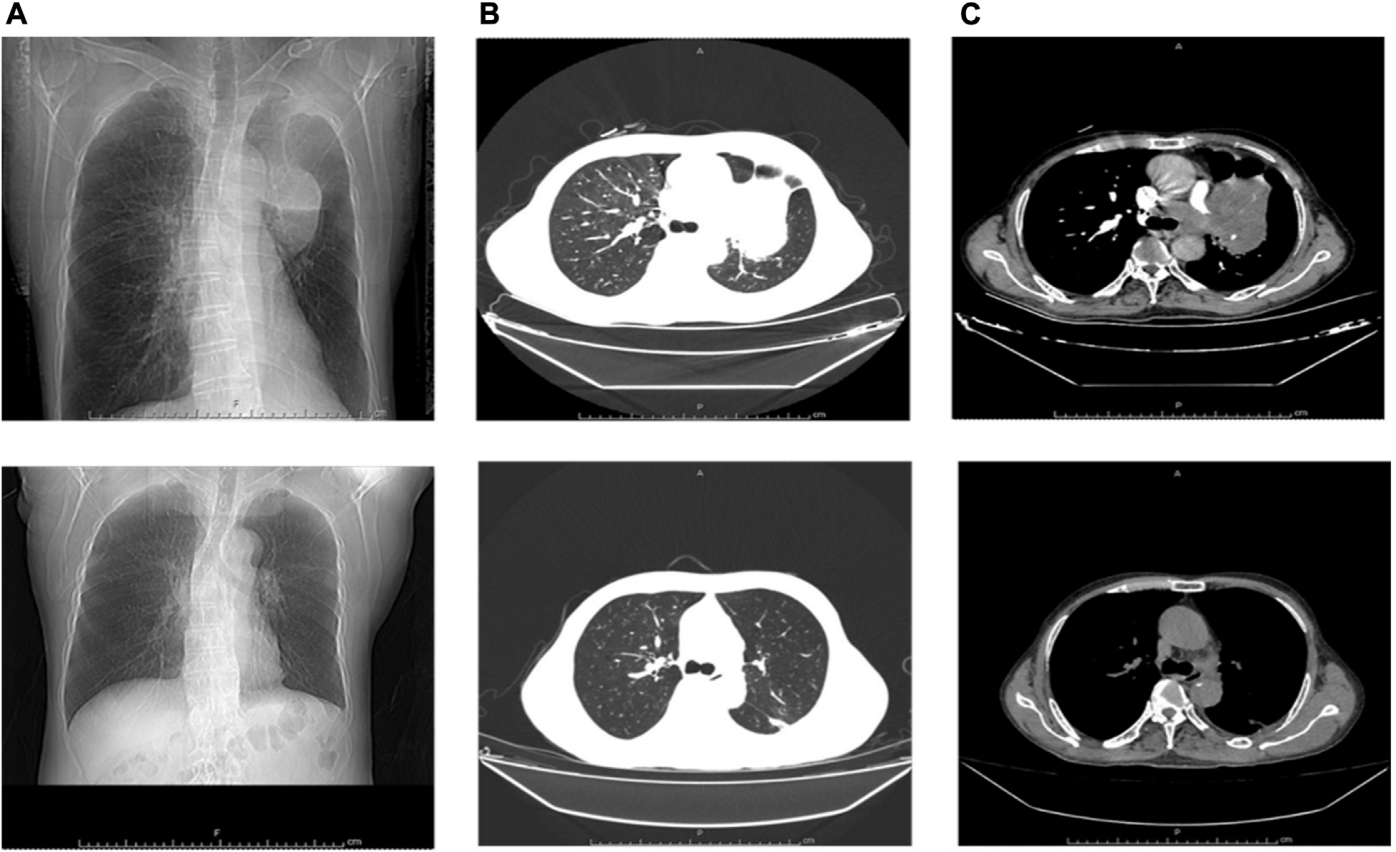
Comparison of lung CT scan before and after chemotherapy. **(A–C)** Before chemotherapy; **(A–C)** After chemotherapy; **(A)**, (a) Frontal; **(B)**, (b) Lung Window; **(C)**, (c) Mediastinum Window.

Therefore, the patient was admitted to our hospital on 24 May (Day 35) with complaints of progressed generalized muscle pain and weakness after chemotherapy for small cell lung cancer. The patient was a smoker but had no history of hypertension, diabetes mellitus, autoimmune disease, or renal disease and no relevant family history. He did not have any history of infection, trauma, hyperthermia, myopathies (myositis, dystrophy, or others) or taking statins, bisphosphonates, antidiabetics, or other drugs prior to admission. He began to have difficulty swallowing food or drinking water and his urine was dark yellow at admission. At that time, his height, weight, blood pressure, heart rate, temperature, respiratory rate, and peripheral capillary oxygen saturation (SpO_2_) were 180 cm, 73 kg, 135/75 mmHg, 71 beats/min, 36.1°C, 20 breaths/min, and 95% on room air, respectively. Laboratory tests on admission showed substantially increased serum CK, Mb, ALT, AST, and LDH (Table 1). In addition, the renal function and myocardial were abnormal: cardiac troponin I (cTnI) 46.5 pg/mL, blood urea nitrogen (BUN) 11.70 mmol/L, serum creatinine (Scr) 105 μmol/L, and uric acid (UA) 508.0 μmol/L. Autoimmune myositis–associated antibodies (anti-Ro-52, anti-Jo-1, anti-Mi-2, anti-Ku, anti-PM-Scl100, anti-PM-Scl75, anti-SRP, anti-PL-7, anti-PL-12, and anti-EJ), vasculitis-associated autoantibodies (anti-pANCA, anti-cANCA, anti-MPO, and anti-PR3), and rheumatism autoantibodies (anti-ANA, anti-dsDNA, anti-ENA, anti-RNP A, anti-RNP 68, anti-Sm/nRNP, anti-Sm, anti-SS-A, anti-SS-B, and anti-Scl-70) were negative and thyroid function tests (thyroid-stimulating hormone and free thyroxine) were normal. In order to exclude infection-induced rhabdomyolysis, IgM antibody tests for pathogens (*mycoplasma* pneumoniae, *chlamydia* pneumoniae, coxsackievirus, adenovirus, respiratory syncytial virus, and cytomegalovirus) were performed and were negative. Surprisingly, a lung CT scan showed that the lung mass was significantly smaller than that before chemotherapy (Figure 1). We recommended a muscle biopsy and electromyography to rule out myopathy; however, this was refused by the patient and his family. According to the Naranjo Scale of adverse drug reactions, the score was 5, which indicates that adverse events were probably adverse drug reactions (Table 2) (Khan et al., 2021). The patient was diagnosed with severe rhabdomyolysis induced by chemotherapy.

**TABLE 1:** Laboratory test results during treatment.

	21 May	24 May	25 May	28 May	31 May	3 Jun	7 Jun	10 Jun
CK (U/L)	11,002	14,947	20,000	15,603	7,516	2,769	1,481	941
ALT (U/L)	165	325	333	274	195	111		
AST (U/L)	518	1,146	1,317	811	473	213		
LDH (U/L)	846	1,236	1,516	1,197	880	653		
BUN (mmol/L)		12.3	11.7	9.7	7.9			
SCr (μmol/L)			105	94	98			
UA (μmol/L)		433.9	508	341	248			

**TABLE 2:** Naranjo adverse drug reaction probability scale.

Question	Yes	No	Do not know	Score
1. Are there previous conclusive reports of this reaction?	+1	0	0	+1
2. Did the adverse event appear after the suspected drug was administered?	+2	−1	0	+2
3. Did the adverse reaction improve when the drug was discontinued or a specific antagonist was administered?	+1	0	0	0
4. Did the adverse event reappear when the drug was re-administered?	+2	−1	0	0
5. Are there alternative causes (other than the drug) that could on their own have caused the reaction?	−1	+2	0	+2
6. Did the reaction reappear when a placebo was given?	−1	+1	0	0
7. Was the drug detected in blood (or other fluids) in concentrations known to be toxic?	+1	0	0	0
8. Was the reaction more severe when the dose was increased or less severe when the dose was decreased?	+1	0	0	0
9. Did the patient have a similar reaction to the same or similar drugs in any previous exposure?	+1	0	0	0
10. Was the adverse event confirmed by any objective evidence?	+1	0	0	0

Note: Adverse Drug Reaction Probability Scale is: Certain, >9; Probable, 5–8; Possible, 1–4; Unlikely, 0.

**FIGURE 2:**
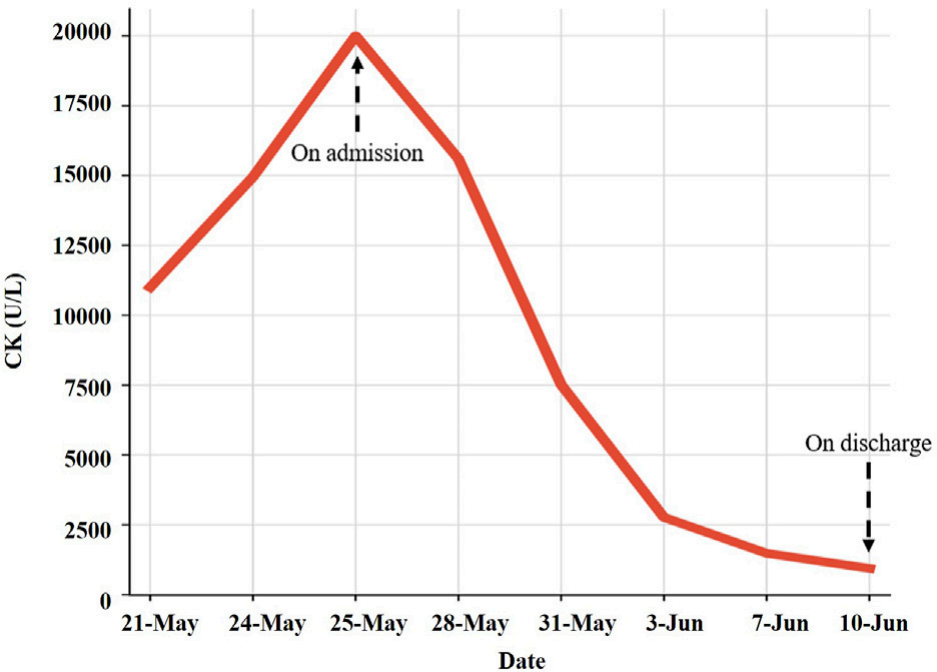
Change of creatine kinase during hospitalization.

**FIGURE 3:**
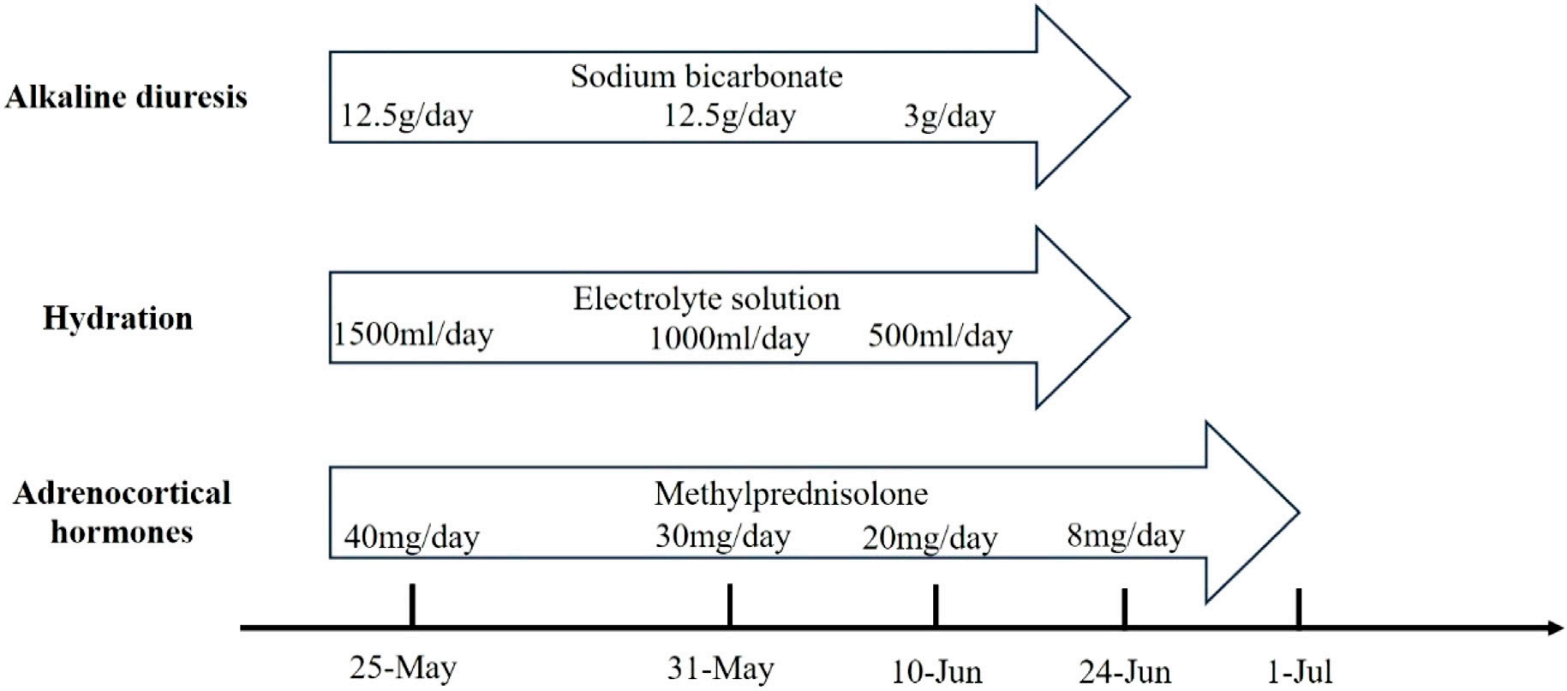
The treatment regime of this patient in 2 months.

Large amounts of intravenous fluids were administered to prevent acute kidney injury and intravenous methylprednisolone (40 mg/day) was started immediately for rhabdomyolysis. Seven days later, intravenous methylprednisolone was changed to 30 mg/day. Serum CK, ALT, AST, and LDH gradually declined within 2 weeks (Table 1). The symptoms of fatigue and myalgia improved. After the 17th day of hospitalization, CK levels decreased to 941 U/L, and the patient requested discharge back to the local hospital for further rehabilitation (Figure 2). Methylprednisolone was continued during the hospitalization and upon discharge. On 10 June (Day 52), the patient was discharged from our hospital with an oral dose of methylprednisolone 20 mg/day, and the dosage was gradually reduced. Serum CK normalized 2 weeks after discharge with an oral dose of methylprednisolone 8 mg/day; however, the symptoms of fatigue and myalgia improved slowly. The treatment regime of this patient within 2 months is shown in Figure 3. After 6 months of follow-up, the patient’s fatigue symptoms still existed and he still refused to receive further anti-tumor drug treatment.

## Discussion

The patient in this case had no previous history of myopathy or immune disease. Liver function, renal function, and electrolyte and urine routines were normal before chemotherapy. After intravenous infusion of etoposide-nedaplatin, the patient gradually developed a general feeling of fatigue, muscle pain, and dark yellow urine. Laboratory tests showed increased serum CK, Mb, ALT, AST, LDH, BUN, Scr, and UA. After excluding trauma, strenuous activity, infections, hyperthermia, and immune factors, the patient was diagnosed with severe rhabdomyolysis induced by chemotherapy. After treatment with intravenous fluids and methylprednisolone, the patient’s symptoms were relieved and laboratory results were significantly improved. Rhabdomyolysis induced by anti-cancer therapy, especially chemotherapy, is still rarely reported. To the best of our knowledge, this is the first case of etoposide-nedaplatin chemotherapy–induced rhabdomyolysis ever reported.

Etoposide, an inhibitor of topoisomerase-II, exerts an anti-cancer effect by inhibiting DNA repair and cell mitosis. It is widely used in treating lung cancer, germ cell tumors, and refractory lymphoma. The main adverse reactions of etoposide include myelosuppression, gastrointestinal symptoms, allergy, alopecia, neurotoxicity, fever, and phlebitis (Khan and Gurav, 2017). Nedaplatin is the second generation of platinum drugs, and is an analog of cisplatin. The anti-cancer mechanism is similar to cisplatin, which inhibits the growth of tumor cells by binding to DNA. It was approved in Japan for use in several cancers, including SCLC (Zhou et al., 2020). It has been allowed to be used in the treatment of esophageal cancer, lung cancer, gynecological cancer, and head and neck tumors in China based on the results of many clinical trials, which showed that nedaplatin has similar anti-tumor therapeutic effects, with a lower toxicity than cisplatin (Ge et al., 2018; Wang et al., 2016; Yang et al., 2012; Zhang et al., 2014). The common adverse effects of nedaplatin include bone marrow suppression, gastrointestinal symptoms, kidney dysfunction, ototoxicity, and alopecia, while allergic reaction and Adams–Stokes syndrome are rare complications (Kawarada et al., 2017).

Although cases of rhabdomyolysis related to etoposide and platinum drugs including carboplatin, cisplatin, and oxaliplatin were reported, no report related to nedaplatin was found. In 1999, Hoshi et al. (1999) reported that a 38-year-old man with choriocarcinoma in the left testis suffered from unconsciousness and hallucinations after receiving high-dose chemotherapy (ifosfamide, carboplatin, and etoposide), and died of respiratory failure. Matsuzaki et al. (2013) reported that a 15-year-old girl with alveolar rhabdomyosarcoma developed severe acute rhabdomyolysis shortly after the second multi-drug chemotherapy cycle, which consisted of etoposide, ifosfamide, actinomycin-D, and vincristine. After mechanical ventilation and fluid replacement, the patient’s condition slowly improved. Sokolova et al. (2017) reported that a 21-year-old man with relapsed non-seminomatous germ cell tumor, who received high-dose chemotherapy with carboplatin and etoposide, presented with bilateral leg pain, and finally improved after treatment. Etoposide combined with platinum drugs is the first-line chemotherapy for small cell lung cancer. To the best of our knowledge, this complication has not been reported with an etoposide-nedaplatin regimen. At present, it is generally believed that the core mechanism of rhabdomyolysis is the decline of intracellular ATP levels and the elevation of myoplasmic Ca^2+^ concentrations (Hohenegger, 2012). Although the mechanism of rhabdomyolysis caused by chemotherapy drugs is not clear, we can speculate on its possible mechanism; that is, chemotherapeutic drugs may damage the muscle membrane, leading to the leakage of cell contents and increased levels of Na^+^ entering the cell. Higher Na^+^ concentrations in cells will activate energy-dependent Na/K ATPase, thus depleting ATP in cells. With the increase of intracellular Na^+^, cells increase the activity of the 2Na^+^/Ca^2+^ exchanger, which removes excess intracellular Na^+^ from the cytoplasm by exchanging it with Ca^2+^, finally increasing the concentration of cytoplasmic calcium (Zhang, 2012). The increased Ca^2+^ concentration activates phospholipase A_2_ and various neutral proteases to degrade cellular phospholipid membranes and various intracellular organelles, which eventually leads to the destruction of muscle cells.

Another unexpected situation is that although the patient had rhabdomyolysis after the first cycle of chemotherapy, the lung mass was obviously reduced on the lung CT scan. One possible hypothesis is that the patient was too sensitive to etoposide or nedaplatin, which led to the simultaneous occurrence of tumor necrosis and muscle injury. However, whether rhabdomyolysis is associated with mass reduction remains uncertain; there is no evidence to support it at present. Certainly, we also consider the possibility of tumor lysis syndrome. Tumor lysis syndrome is characterized by a massive cytolysis, with the release of intracellular electrolytes, nucleic acids, and metabolites into the circulation, resulting in hyperuricemia, hyperkalemia, hyperphosphatemia, hypocalcemia, and even acute renal failure. However, the presented case developed chest tightness, weakness, asthma, and palpitation after Day-2 chemotherapy. Laboratory examination showed that serum UA 369.0 μmol/L, calcium (Ca) 2.22 mmol/L, potassium (K) 4.01 mmol/L, and phosphate (P) 1.24 mmol/L were within normal levels at that time. Serum UA (302.0 μmol/L), Ca (2.20 mmol/L), K (4.33 mmol/L), and P (0.90 mmol/L) were also within normal levels on the third day after chemotherapy. After discharge, the patient developed progressive generalized muscle pain and weakness. Serum CK, Mb, ALT, AST, and LDH levels were significantly increased, while serum UA (341.0 μmol/L), Ca (2.34 mmol/L), and K (4.31 mmol/L) were still within normal levels on this admission. Therefore, it is impossible to diagnose tumor lysis syndrome.

## Conclusion

In conclusion, severe rhabdomyolysis can lead to acute renal failure or even death. Rhabdomyolysis induced by anti-cancer drugs, especially chemotherapy drugs, is rarely reported and easily overlooked. Here, we report the first case of etoposide-nedaplatin chemotherapy–induced severe rhabdomyolysis. It is tempting to speculate that the patient is too sensitive to etoposide or nedaplatin, which would lead to the simultaneous occurrence of tumor necrosis and muscle injury. However, the cause of etoposide-nedaplatin–induced severe rhabdomyolysis is still unclear and more cases need to be collected for analysis in the future. Therefore, we think that physicians should be aware of this rare but potentially serious complication when using chemotherapy drugs. When progressive fatigue and muscle pain occur in patients undergoing chemotherapy, the possibility of acute rhabdomyolysis should be considered. Once the diagnosis is confirmed, the medication should be discontinued immediately and treatment should be carried out, especially to maintain kidney function.

## Data Availability

The raw data supporting the conclusion of this article will be made available by the authors, without undue reservation.
